# Metastable dynamics in heterogeneous neural fields

**DOI:** 10.3389/fnsys.2015.00097

**Published:** 2015-06-30

**Authors:** Cordula Schwappach, Axel Hutt, Peter beim Graben

**Affiliations:** ^1^Department of German Studies and Linguistics, Humboldt-Universität zu BerlinBerlin, Germany; ^2^Department of Physics, Humboldt-Universität zu BerlinBerlin, Germany; ^3^Team Neurosys, InriaVillers-les-Nancy, France; ^4^Team Neurosys, Centre National de la Recherche Scientifique, UMR nō 7503, LoriaVillers-les-Nancy, France; ^5^Team Neurosys, UMR nō 7503, Loria, Université de LorraineVillers-les-Nancy, France; ^6^Bernstein Center for Computational Neuroscience, Humboldt-Universität zu BerlinBerlin, Germany

**Keywords:** neural fields, kernel construction, metastability, heteroclinic orbits, trial-to-trial variability, distributed representations, sub-networks, sparsity

## Abstract

We present numerical simulations of metastable states in heterogeneous neural fields that are connected along heteroclinic orbits. Such trajectories are possible representations of transient neural activity as observed, for example, in the electroencephalogram. Based on previous theoretical findings on learning algorithms for neural fields, we directly construct synaptic weight kernels from Lotka-Volterra neural population dynamics without supervised training approaches. We deliver a MATLAB neural field toolbox validated by two examples of one- and two-dimensional neural fields. We demonstrate trial-to-trial variability and distributed representations in our simulations which might therefore be regarded as a proof-of-concept for more advanced neural field models of metastable dynamics in neurophysiological data.

## 1. Introduction

Metastable states and transient dynamics between metastable states have received increasing interest in the neuroscientific community in recent time. Beginning with Dietrich Lehmann's original idea to identify “atoms of thought” as metastable topographies, so-called *brain microstates*, in spontaneous and event-related electroencephalograms (EEG) (Lehmann et al., 1987; Lehmann, 1989; Lehmann et al., 2009), experimentalists found accumulating evidence that metastability is tentatively an important organization principle in neurodynamical systems. Mazor and Laurent (2005), e.g., reported metastable states in the locust odor system (cf. Rabinovich et al., 2001, 2008a), while Hudson et al. (2014) found metastability in the local field potentials of rats recovering from anesthesia. For the analysis of human EEG, several segmentation techniques into metastable states have recently been suggested by Hutt (2004), Allefeld et al. (2009), and beim Graben and Hutt (2015).

From a theoretical perspective, metastable EEG topographies or components of the event-related potential (ERP) have been identified with saddle-nodes in deterministic low-dimensional systems by Hutt et al. (2000) and Hutt and Riedel (2003). Particularly, the discoveries of winnerless competition (Rabinovich et al., 2001; Seliger et al., 2003) and heteroclinic orbits in neural population dynamics (Afraimovich et al., 2004a,b; Rabinovich et al., 2008b) led to better understanding of metastability and transient behavior in theoretical neuroscience. Winnerless competition is ubiquitous in complex excitation-inhibition networks with strong asymmetries. While symmetric connectivity usually leads to Hopfield-type attractor neural networks (Hopfield, 1982; Hertz et al., 1991) where transient dynamics is only observed for the motion from a basin of attraction toward an asymptotically stable fixed point attractor, winnerless competition between neural Lotka-Volterra populations (Fukai and Tanaka, 1997; Cowan, 2014) allows for hierarchical transient computations, bifurcations, and the resolution of sequential decision problems, as applied for modeling speech processing (Kiebel et al., 2009), bird songs (Yildiz and Kiebel, 2011), syntactic parsing (beim Graben and Potthast, 2012), and, most recently, working memory (Rabinovich et al., 2014a,b).

However, these phenomena have been investigated on the rather abstract level of macroscopic neural populations so far, without reference to the mesoscopic and microscopic levels of spatially given nervous tissue and individual neurons. One important approach to characterize the former, nervous tissue at the mesoscopic scale, are *neural fields*, i.e., continuum approximations of infinitely large neural networks (Coombes et al., 2014). In a recent theoretical study, beim Graben and Hutt (2014) investigated stationary states and heteroclinic dynamics in neural fields with heterogeneous synaptic connectivity. The present work applies this previous study to describe experimentally observed transient neural activity as a proof-of-concept of our theoretical approach. We propose a novel hypothesis on the origin of trial-to-trial variability observed in most experimental data, on episodic cell assembly dynamics and on sparsely sampled neural representations.

Moreover, we disseminate our software implementation as a MATLAB *neural field toolbox* to facilitate further research on this intriguing field of computational neuroscience.

## 2. Materials and methods

In this section we present some of the theoretical findings of beim Graben and Hutt (2014) and indicate how they have been implemented in our simulations.

### 2.1. Theoretical background

An important representative of neural fields is given through the *Amari equation*

(1) $$\frac{\partial u(x,t)}{\partial t}=-u(x,t)+\int_{\Omega }w(x,y)\;f(u(y,t))\text{ d}y$$

describing the evolution of neural activity *u*(*x*, *t*) at site *x* ∈ Ω ⊂ ℝ^*d*^ and time *t* (Amari, 1977). Here, Ω is a *d*-dimensional manifold, representing neural tissue. Moreover, *w*(*x*, *y*) is the synaptic weight kernel, and *f* is a sigmoidal activation function, usually chosen as *f*(*u*) = 1/(1 + exp(−β (*u* − θ))), with gain β > 0, and threshold θ > 0. The time scale of the dynamics, often characterized by a particular time constant is implicitly included in the kernel *w*(*x*, *y*).

The neural field described by Equation (1) is called homogeneous when the kernel is translation invariant: *w*(*x*, *y*) = *w*(*x* − *y*). If the field is not homogeneous it is called heterogeneous.

Stationary states, *v*(*x*), of the Amari equation which are obtained from ∂*u*/∂*t* = 0 obey the nonlinear Hammerstein integral equation

(2) $$v(x)=\int_{\Omega }w(x,y)f(v(y))\text{ d}y\;.$$

By choosing a heterogeneous *Pincherle-Goursat kernel* (Veltz and Faugeras, 2010)

(3) w(x,y)=v(x)v(y),

and carrying out a linear stability analysis, beim Graben and Hutt (2014) were able to prove that the stationary state *v*(*x*) is either an asymptotically stable fixed point attractor, or a saddle with a one-dimensional unstable manifold, i.e., a metastable state. Since such saddles could be connected along their stable and unstable directions, heterogeneous neural fields may exhibit stable heteroclinic sequences (SHS: Afraimovich et al., 2004b; Rabinovich et al., 2008b).

Let {*v*_*k*_(*x*)}, 1 ≤ *k* ≤ *n* be such a collection of metastable states which we assume to be linearly independent. Then, this collection possesses a biorthogonal system of adjoints {*v*^+^_*k*_(*x*)} obeying

(4) $$\int_{\Omega }v_{j}^{+}(x)v_{k}(x)\text{ d}x=\delta_{jk}\;.$$

For the particular case of Lotka-Volterra neural populations, described by activities ξ_*k*_(*t*),

(5) $$\frac{\text{d}\xi_{k}}{\text{d}t}=\xi_{k}\left(\sigma_{k}-\sum_{j=1}^{n}\rho_{kj}\xi_{j}\right)$$

with growth rates σ_*k*_ >0, interaction weights ρ_*kj*_ >0 and ρ_*kk*_ = 1 that are tuned according to the algorithm of Afraimovich et al. (2004b) and Rabinovich et al. (2008b), the population amplitude

(6) $$\alpha_{k}(t)=\frac{\xi_{k}}{\sigma_{k}}$$

recruits its corresponding metastable state *v*_*k*_(*x*), leading to an order parameter expansion

(7) $$u(x,t)=\sum_{k=1}^{n}\alpha_{k}(t)v_{k}(x)$$

of the neural field.

Under these assumptions, beim Graben and Potthast (2012) and beim Graben and Hutt (2014) have explicitly constructed the kernel *w*(*x*, *y*) through a power series expansion of the right-hand-side of the Amari equation (Equation 1),

(8) $$\begin{array}{l} \frac{\partial u(x,t)}{\partial t}=-u(x,t)+\int_{\Omega }w_{1}(x,y)u(y,t)\text{ d}y\\ \;+\int_{\Omega }\int_{\Omega }w_{2}(x,y,z)u(y,t)u(z,t)\text{ d}y\text{ d}z \end{array}$$

with Pincherle-Goursat kernels^1^.

(9) $$w_{1}(x,y)=\sum_{k}\left(\sigma_{k}+1)v_{k}(x)v_{k}^{+}(y\right)$$

(10) $$w_{2}(x,y,z)=-\sum_{kj}\sigma_{j}\rho_{kj}v_{k}(x)v_{k}^{+}(y)v_{j}^{+}(z)\;.$$

Interestingly, the kernel *w*_1_(*x*, *y*) describes a Hebbian synapse between sites *y* and *x* whereas the three-point kernel *w*_2_(*x*, *y*, *z*) further generalizes Hebbian learning to interactions between three sites *x*, *y*, *z* of neural tissue.

### 2.2. Numerical studies

For a numerical implementation of the theoretical results above, we have to discretize time and space. Using MATLAB, temporal discretization on the one hand is achieved through the ordinary differential equation solver ode15s for stiff problems. On the other hand, spatial discretization converts the kernels *w*_1_ and *w*_2_ into tensors of rank two and three, respectively. Consequently, the integrals in Equation (8) become contractions over products of tensors and state vectors *u*(*t*). In order to properly deal with tensor algebra, we use the Sandia Tensor Toolbox^2^. Our neural field toolbox, thus obtained is available as Supplementary Material. We evaluate our implementation in the next subsections by means of two examples.

#### 2.2.1. One-dimensional neural field

In our first simulation, we use a *d* = 1 dimensional neural field where we choose *n* = 3 sine functions

(11) $$v_{k}(x)=\sin kx$$

as metastable states on the domain Ω = [0, 2π] discretized with a spatial grid of *N*_*x*_ = 100 sites. According to the orthogonality relations

(12) $$\int_{\Omega }\sin jx\sin kx\text{d}x=\pi \delta_{jk}$$

we easily obtain the adjoint modes

(13) $$v_{k}^{+}(x)=\frac{1}{\pi }\sin kx\;.$$

**Figure 1:**
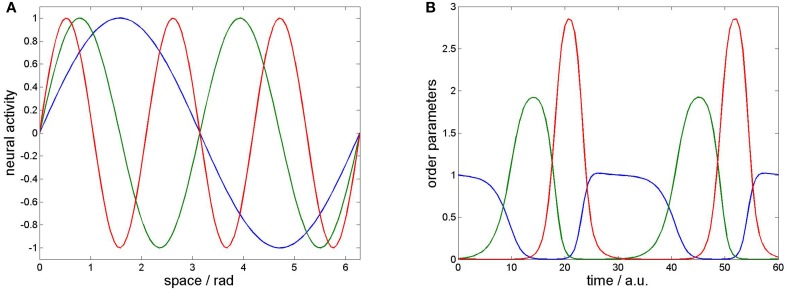
**Prescribed dynamics. (A)** Three sinusoids as spatial patterns. **(B)** Stable heteroclinic contour resulting from winnerless competition in a Lotka-Volterra system (Equation 5). Blue: *k* = 1, green: *k* = 2, red: *k* = 3.

For the temporal dynamics we prepare the stable heteroclinic contour solving (Equation 5) used by beim Graben and Hutt (2015) with σ_1_ = 1, σ_2_ = 2, σ_3_ = 3. Metastable states *v*_*k*_(*x*) and their population activities ξ_*k*_(*t*) are shown in Figure 1.

We run simulations with one fixed initial condition and also from an ensemble of 60 initial conditions randomly distributed in the vicinity of the first saddle, where we add some small portion of Gaussian observational noise (noise level σ = 0.005) in order to demonstrate trial-to-trial variability and hence event-related phase decoherence (Jung et al., 2001; Makeig et al., 2002).

#### 2.2.2. Two-dimensional neural field

For our second demonstration, we assume a spatially distributed response in a neural population to external stimuli triggering a sequence of neural activity patterns. It is well-established that sensory input features (Pasupathy and Connor, 2002) at earlier stages of the object's representation pathway and memory (Rissman and Wagner, 2012) is encoded by distributed cortical neural populations while objects are sparsely coded in later stages of the representation pathway (Connor, 2005). Here we consider a cortical neural population embedded in two-dimensional space involving interleaved patterns. These patterns are *d* = 2 dimensional gray scale bitmap images of the numbers^3^ 1, 2, and 3 (see **Figure 4** in Section 3.2). In the implementation, these bitmaps are downsampled to a 20 × 20 grid and reshaped into vectors with *N*_*x*_ = 400 elements. Adjoint patterns are obtained as Moore-Penrose pseudoinverses (Hertz et al., 1991).

The temporal evolution of these patterns follows the same heteroclinic contour as above. Here, the underlying working assumption is the presence of interacting sub-networks, e.g., reflecting several distributed representations of signal features or of pieces of working memory. The study predicts what one expects to measure in single spatial locations while the neural system encodes information in a spatially distributed population.

## 3.1. Results

The results of our simulation studies are presented in this section.

### 3.2. One-dimensional neural field

**Figure 2:**
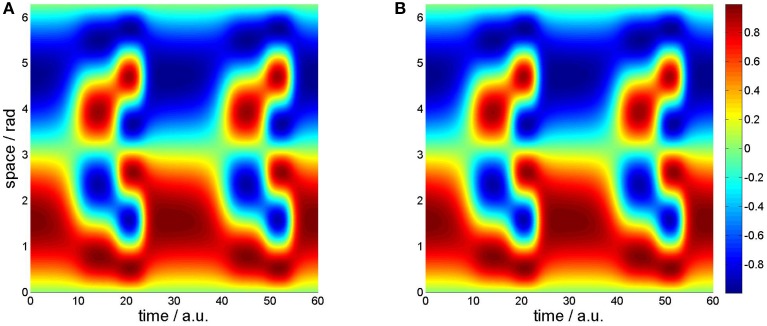
**One-dimensional spatiotemporal dynamics. (A)** Prescribed trajectory from order parameter ansatz (Equation 7). **(B)** Solution of the Amari (Equation 8).

For the one-dimensional neural field we compare in Figure 2 the prescribed spatiotemporal dynamics as resulting from the order parameter expansion (Equation 7) with the solution of the Amari (Equation 8).

Figure 2A shows the prescribed dynamics on a spatiotemporal grid with time on the x-axis and space on the y-axis. The instantaneous activations are therefore given by vertical slices. Going from left to right, these slices first exhibit one wave crest (in red at the bottom) and one wave trough (in blue at the top), corresponding to metastable state *v*_1_(*x*). Around time *t* = 15 the frequency doubles and metastable state *v*_2_(*x*) can be observed for approximately seven ticks. The third metastable state met by the trajectory around time *t* = 21 is the mode with tripled frequency. It is only stable for five ticks and evolves thereafter into the first mode again.

In contrast, Figure 2B depicts the numerical solution of the Amari equation (Equation 8). Obviously, no deviation is visible.

In order to draw neurophysiologically relevant conclusions from our toy model, we consider the metastable states of the heteroclinic contour as “synthetic ERP components” (Barrès et al., 2013) measured with “electrodes” at the particular sampling points. Because ERPs are obtained from averaging spontaneous EEG over ensembles of several trials that are time-locked to the perception or processing of stimuli, we simulate 60 synthetic ERP trials by randomly preparing initial conditions of the Amari equation.

**Figure 3:**
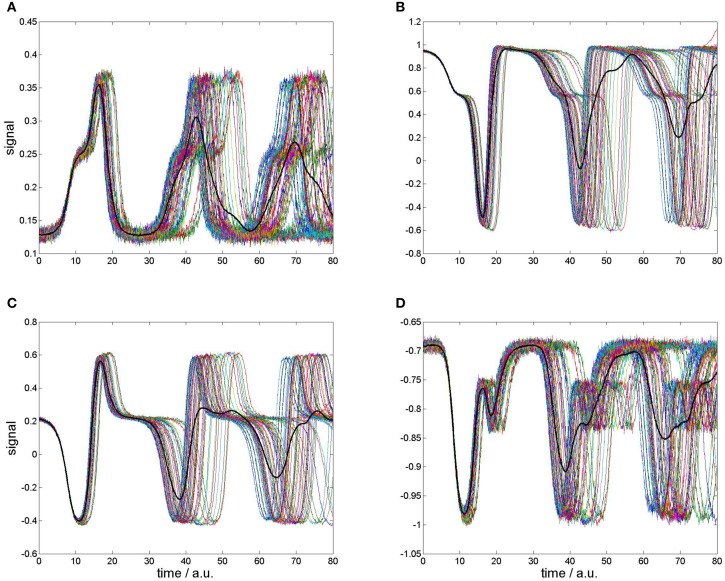
**Four selected “recording sites” for neural field simulation with 55 randomly prepared initial conditions (colored traces) and “grand average” (bold black trace). (A)** At position: 3, **(B)** position 21, **(C)** position 47, **(D)** position 88.

The results are displayed in Figure 3 for four “measurement electrodes” at positions 3, 21, 47, and 88. Interestingly, our algorithm exhibited numerical instabilities in five runs which have been marked as “rejected” outliers and excluded from presentation. The resulting 55 trials are shown as colored traces in Figure 3. At simulation start all signals are nicely coherent, but later substantial phase dispersions take place (Jung et al., 2001; Makeig et al., 2002).

We also calculated the ERP averages from our simulation shown as bold black traces in Figure 3. On the one hand, the averaged ERP is much smoother than the noisy single realizations which justifies averaging in our simulation. However, the averaged ERP significantly decays in the course of time. This is obviously due to the increasing phase decoherence (Jung et al., 2001; Makeig et al., 2002).

### 3.2. Two-dimensional neural field

**Figure 4:**
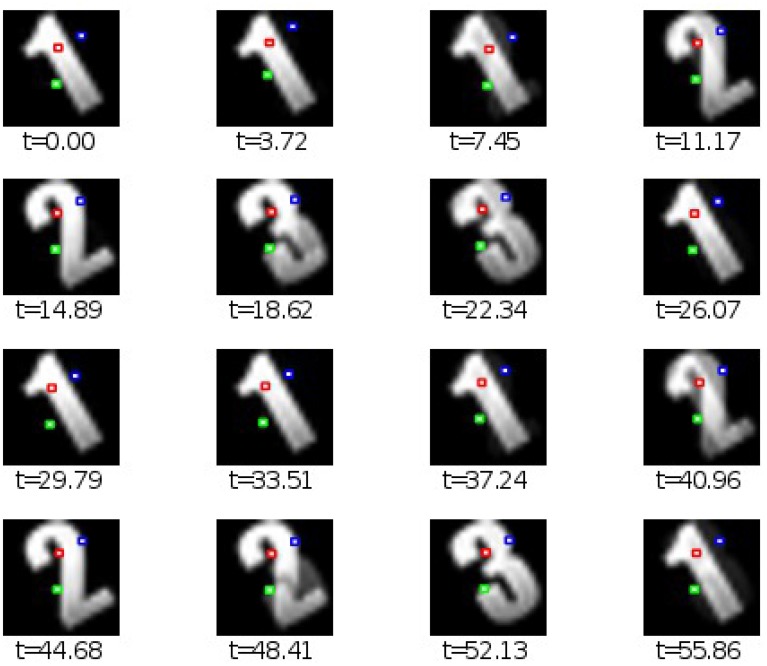
**Two-dimensional spatiotemporal solution of model (Equation 9) considering spatial patterns of the numbers “1,” “2,” and “3” as spatial modes** ***v*****_1_(*x*), *v*_2_(*x*), and** ***v*****_3_(*x*), respectively**. The three color-coded points denote three spatial locations whose temporal evolution is shown in **Figure 5**.

The numerical simulation of Equation (8) yields a sequence of two-dimensional transient patterns which is shown as a sampled sequence of snapshot maps in Figure 4.

According to the different growth rates σ_*k*_ of the populations, pattern “1” stays the longest period of time, pattern “2” is visible for a shorter period of time and pattern “3” can be seen for the shortest period of time. These modes represent interweaved spatial networks reflecting intrinsically stored activity patterns.

**Figure 5:**
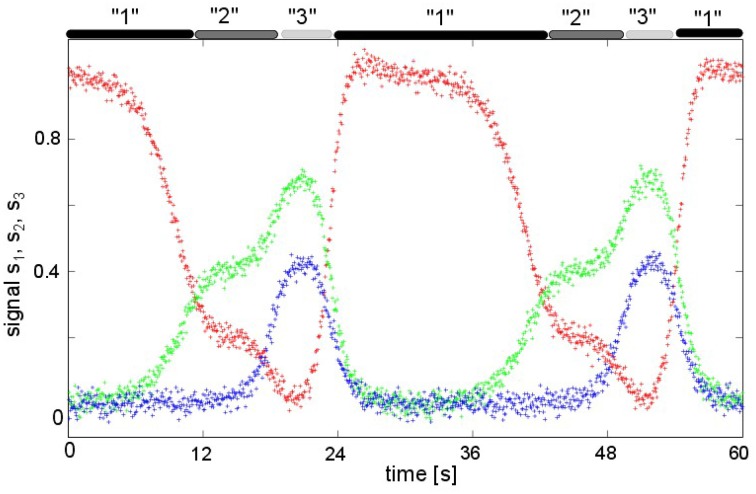
**The time-dependent activity** ***u*****(*****x***_***l***_**,** ***t*****) at three spatial locations** ***x***_***l***_**,** ***l*** **= 1, 2, 3 defined in Figure 4**. The upper gray-colored bars denote the emergence time intervals of the corresponding patterns in Figure 4. The color codes of the time series correspond to the respective colors of the spatial locations in Figure 4.

Now assuming that measurement of neural activity takes place at discrete spatial locations (color-coded points in Figure 4), one observes different transient dynamics dependent on the spatial location of the measurement point that is shown in Figure 5. Considering the red-coded spatial location, one observes strong activity in the time periods when pattern “1” is active, and well-reduced activity in the time windows of active patterns “2” and “3.” Conversely, the activity at the blue-coded location defined in Figure 4 raises only if pattern “3” is active, otherwise its activity is well-reduced. The green-coded spatial location shows negligible activity in time periods when pattern “1” is active while activity is increased during the emergence of patterns “2” and “3.”

## 4. Discussion

In this paper, we presented a software implementation (*neural field toolbox*) and numerical simulation results of previously reported theoretical findings on metastable states and heteroclinic dynamics in neural fields (beim Graben and Potthast, 2012; beim Graben and Hutt, 2014). For the particular case of Lotka-Volterra population dynamics and linearly independent spatial modes, the synaptic weight kernel of the Amari neural field equation (Amari, 1977) can be explicitly constructed from the prescribed metastable states and their evolution parameters as Pincherle-Goursat kernels. This is an important finding as our kernel construction method is not a standard training algorithm such as backpropagation (Igel et al., 2001; beim Graben and Potthast, 2009). Yet it implements a straightforward generalization of Hebbian learning algorithms (beim Graben and Potthast, 2009; Potthast and beim Graben, 2009).

We validated our algorithm by means of two examples, a one-dimensional neural field where metastable states are three sinusoidal excitations over a line, and a two-dimensional example where we have chosen three bitmap images as spatial modes. The temporal dynamics was prescribed as a heteroclinic contour connecting these three patterns in a closed loop. In both simulations, the results were in exact agreement with the prescribed trajectories.

Furthermore, we examined the issues of trial-to-trial variability and distributed representations. In the first example we created solutions for randomly prepared initial conditions, thereby emulating phase resetting in event-related brain potentials (ERP). We observed increasing phase decoherence in the resulting ERP averages. Our model presents a theoretically satisfying explanation for this ubiquitous experimental finding (Jung et al., 2001; Makeig et al., 2002). Assuming that ERP components are metastable states that are connected along heteroclinic orbits (Hutt and Riedel, 2003; beim Graben and Hutt, 2015), single ERP trials start from randomly distributed initial conditions, sometimes closer and sometimes farther from the respective metastable stable. These initial distances from a metastable state result in acceleration and hence in velocity differences in phase space, eventually leading to dispersion and decoherence. Moreover, such a dependence on initial conditions resembles previous experimental results by Pastalkova et al. (2008) showing that identical experimental initial conditions in a motor task lead to identical sequences of cell assembly activations, while different initial conditions yield different sequences.

For the second example we considered the interaction of three two-dimensional populations, cf. Figure 4. The transient passage of the system at metastable attractors has been shown experimentally in previous studies, such as in middle-latent auditory evoked potentials (Hutt and Riedel, 2003) or in the population response of olfactory projection neurons to odor stimuli (Mazor and Laurent, 2005). For instance, the study of Mazor and Laurent (2005) also shows nicely the responses of single neurons in the population revealing different activity in different neurons: some neurons respond to the external stimulus, others remain silent. Such a distinction in response can easily be explained by an insufficient spatial sub-sampling in the measurement and the presence of spatially distributed patterns. However, just spatial sub-sampling does not explain the fully distinct activity of different neurons, such as different episode neurons found in the hippocampus (Pastalkova et al., 2008). Here, different neurons show distinct episodic temporal activities. The equivalent temporal evolution is shown in our simulations in Figure 5, where the units at different spatial locations exhibit different temporal sequences of activation that are highly correlated to the presence of the respective pattern representations. This difference results from interacting populations or cell assemblies.

The latter line of argumentation raises the question whether it may explain previous results on sparse neural representations or even may contribute to the question on the existence of “grandmother cells” (Connor, 2005; Quiroga et al., 2005). At a first glance, the present work assumes the existence of interacting spatially distributed sub-networks and supports their existence by a qualitative comparison to previous experimental results by Mazor and Laurent (2005) and Pastalkova et al. (2008). Our assumption of interacting sub-networks does not rule out sparse neural representations since our modeling approach does not stipulate contiguous spatial patterns but also allows for sparse patterns as well.

Metastable neural field dynamics as an ubiquitous organization principle of the brain is also consistent with findings from neuroanatomy and cognitive neuroscience. Anatomically, neural circuits comprise convergent and divergent pathways between populations (Kandel et al., 1991). Assuming that a particular sub-network gets activated by percolation along a convergent pathway and deactivated along a divergent pathway subsequently entails a saddle-node picture in its phase space description, hence a metastable attractor. In cognitive neuroscience, mental representations are regarded as intermediate results of cognitive computations in discrete time. In order to embed these into continuous physical time, they have to be considered connected through continuous trajectories along their stable and unstable directions, i.e., as metastable states, again (beim Graben and Potthast, 2009, 2012).

The present study is a first step toward metastability in neural fields. We hope that our work encourages further research on metastability in neural fields to describe transient neural dynamics by interacting populations and contribute to the description of neural information storage, being either distributed or sparse.

## Author contributions

This study reports results from CS's student internship at Department of German Studies and Linguistics, Humboldt-Universität zu Berlin. CS developed the program code and conducted the numerical simulations. AH included the sub-network study, PbG contributed the study on trial-to-trial variability and compiled the neural field toolbox. All authors wrote the manuscript together.

## Funding

PbG acknowledges support by a Heisenberg Fellowship of the German Research Foundation DFG (GR 3711/1-2) and of the Bernstein Center for Computational Neuroscience, Berlin, hosting AH as visiting professor during October 2014. AH acknowledges funding from the European Research Council for support under the European Union's Seventh Framework Programme (FP7/2007-2013)/ERC grant agreement no. 257253.

### Conflict of interest statement

The authors declare that the research was conducted in the absence of any commercial or financial relationships that could be construed as a potential conflict of interest.

## Footnotes

^1^There was a mistake in our previous reports (beim Graben and Potthast, 2012; beim Graben and Hutt, 2014). Although the kernel construction has been correctly derived, a minus sign was omitted in the final result for kernel *w*_2_(*x*, *y*, *z*). This is corrected now.

^1^http://www.sandia.gov/~tgkolda/TensorToolbox/index-2.5.html

^2^Original images are taken from the webpage http://www.iconarchive.com/tag/number-3 before modifications with respect to color and resolution.

## Supplementary material

### Supplemental data

A GIF animation of the two-dimensional neural field simulation is given as Supplementary Material. Moreover, we deliver a MATLAB software neural field toolbox. This package essentially comprises three routines: amarikernels, amarieq, iniamari, and a main program, solveamari to be evoked in the the following way: amarikernels is the training program for the synaptic weights. It takes four arguments: *V*_*patterns*, *sigmarange*, *compbias*, and *contourflag* and returns two kernel tensors *K*1, *K*2. *V*_*patterns* is a matrix whose columns are the metastable states in a spatial discretization, their order corresponds to the desired heteroclinic sequence. *sigmarange* is an interval of Lotka-Volterra grow rates σ characterizing the time scale of the dynamics, while *compbias* denotes the competition bias in the interaction matrix (ρ). The last parameter, *contourflag*, is a binary flag deciding whether the hereroclinic sequence is closed (1) or not (0). A closed hereroclinic sequence is called hereroclinic contour. amarieq defines the Amari equation (Equation 8) for the ODE solver. It has four input arguments, *t*, *V*, *K*1, *K*2, where *t* is the time span to be simulated, *V* is the actual field activity, and *K*1, *K*2 are the two synaptic weight kernels. iniamari prepares an initial condition at the surface of the simplex spanned by the metastable states *V*_*patterns*. The other arguments are *lead* and *remain*, denoting the leading direction toward the next saddle and its orthogonal projection on the remaining modes. Finally, solveamari presents the code for our one-dimensional neural field example, evoking the ODE solver in line 48.
