# Supplementary figures and images for: Metastable dynamics in heterogeneous neural fields
Source: Front Syst Neurosci. 2015 Jun 30;9:97. doi: 10.3389/fnsys.2015.00097 (PMC4485166; doi:10.3389/fnsys.2015.00097)

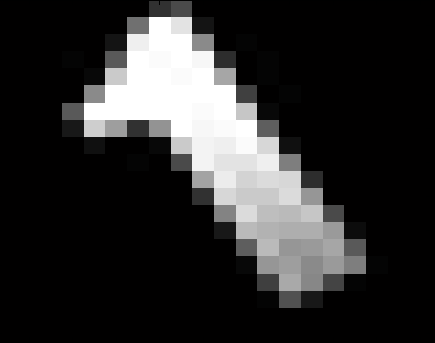

Supplement: Supplementary file 1 [file Presentation1.ZIP › fig4anime.gif]
